# Psychological Quarterly Retrospect

**Published:** 1856-10-01

**Authors:** 


### *Psychological Quarterly Retrospect.*

Our last quarterly retrospect was omitted. So plentiful are the subjects that present themselves to our notice this quarter that we have only to stop and gather them as we pass, and the difficulty, if any, rather lies in the selection of our materials, than in the want of them. The daily press collects everything. Little and great, the grand and the insignificant, are equally to be found recorded in its columns; and we live in an age when the passing events of the world range themselves, almost like words, under their proper letters in a vast index, as easy of access as they are of value to every class of enquirers.

We have been much struck with the following affecting account (extracted from Lord John Russell's Life and Correspondence of the Poet) of the last days of the author of the Irish Melodies.

"Moore's sun set in gloom; and the years which closed his chequered career were clouded, not only with pecuniary embarrassments, but with such dismal events as the death of his two sons, which left him in the melancholy position of surviving his five children.

"The death of his only remaining child, and his last and most beloved sister, deeply affected the health, crushed the spirits, and impaired the mind of Moore. An illness of an alarming nature shook his frame, and for a long time made him incapable of any exertion. When he recovered, he was a different man. His memory was perpetually at fault, and nothing seemed to rest upon his mind. He made engagements to dinners and parties, but usually forgot half of them. When he did appear, his gay flow of spirits, happy application of humorous stories, and constant and congenial ease, were all wanting. The brilliant hues of his varied conversation had failed, and the strong powers of his intellect had manifestly sunk. There was something peculiarly sad in the change. It is not unusual to observe the faculties grow weaker with age; and in the retirement of a man's own home, there may be 'no unpleasing melancholy' in the task of watching such a decline. But when in the midst of the gay and the convivial, the wit appeared without his gaiety, and the guest without his conviviality—when the fine fancy appeared not so much sobered as saddened, it was a cheerless sight.

"Moore's last days were calm and peaceful. His domestic sorrows, his literary triumphs, seem to have faded away alike into a calm repose. He retained to his last moments a pious submission to God, and a grateful sense of the kindness of her whose tender office it was to watch over his decline."

The *Edinburgh Review*, for July, 1856, gives a similar picture of an acquaintance, if not the companion, of Moore:—

"Till near ninety, Rogers was a striking exception to the rule 'of the decay of the mind before that of the body.' He then gradually dropped into that state, mental and bodily, which raises a reasonable doubt whether prolonged life be a blessing or a curse—

'Omni
Membrorum damno major dementia, quæ nec
Nomina servorum, nec vultus agnoscit amicûm,
Cum queis præteritâ cœnavit nocte, nec illos
Quos genuit, quos eduxit.'

Although his impressions of long past events were as fresh as ever, he forgot the names of his relations and oldest friends, whilst they were sitting with him, and told the same stories to the same people, two or three times over in the same interview. But there were frequent glimpses of intellect in all its original brightness, of tenderness, of refinement, and of grace. 'Once driving out with him,' says a female correspondent, 'I asked him after a lady whom he could not recollect. He pulled the check-string, and appealed to his servant. 'Do I know Lady M——?' The reply was, 'Yes, sir.' This was a painful moment to us both. Taking my hand, he said, 'Never mind, my dear, I am not yet reduced to stop the carriage and ask if I know you.' "

A friend of the Editor once ventured to ask Rogers if he remembered what Lord Byron had said after visiting his house and admiring the choiceness of its style. "What must this man not have suffered to have accomplished all this?" "It is perfectly true," replied Mr. Rogers, pinching up his skinny cheeks between his forefinger and thumb; "it is perfectly true—they have left me nothing but this!" The anecdote, thus related, took place at the London Institution, in the private library of his friend Mr. Maltby, with whom the party alluded to was well acquainted—it was in the year 1834 or 1835. Mr. Maltby himself, the clever connoisseur of a title-page, and a bookworm of no mean note, died at the advanced age of ninety, feeble, but in the possession of his faculties.

Humboldt is an instance of intellect undecayed by age. Strabo wrote his Geography, it is said, at eighty-two; and Michael Angelo, who died at eighty-eight, preserved his mind and genius to the last. His last will and testament was as grand as it was laconic, while critics are disposed to consider his last productions better than his first. On the other hand, the brightest efforts of genius have been conceived and executed before the meridian of life; of which Byron, Scott, Pope the poet, Mozart, Weber, Tasso, Shakespeare, Sir Isaac Newton and others, are illustrious examples. It is popularly supposed, that Homer composed his immortal epic in advanced life, and in painting and statuary he is usually represented as the blind old bard. Yet this was not the case. Perhaps the mistake arose from the Homer who recited those wonderful verses to his admiring hearers not being *the* Homer who had composed them. It is the opinion of the master critic, Longinus, that the *Iliad* was the production of a mind in the vigour of manhood, and the *Odyssey* the poetic recreation or repetition of the evening of life. We agree with the great Longinus. For there are, as he says, some puerilities in the *Odyssey*, while there are none in the

*Iliad;* and the order of events forbids the conjecture that the latter was composed before the former; and it must be owned, that with all its quiet beauties, the *Odyssey* wants the pathos, the depth of colouring, the majestic ease and force of the *Iliad.*

The preservation of the intellect to the latest period of age depends upon circumstances, over many of which we have no control. The nerves may be weak by nature, or accidentally decay the first; or there may be a scrofulous or gouty taint, the heirloom of the family; or a failure in the functions of the heart or stomach, natural or acquired. The early part of life may have been corroded by anxiety, weakened by privations, or overstrained by toil, which neither we nor our progenitors could either foresee or prevent. Wine or ardent spirits may have been too freely indulged in, and their use apologized for upon the plea of social engagements or a feeble constitution; while the more sensual passions may not have been held in with the curb of a tightened rein. Fortune may have arrived when she has ceased to be sought for, and reputation or celebrity bestowed or achieved when it is too late to facilitate the happiness of ourselves, and more especially of those with whom we are surrounded. In each of these instances, the mind decays early, and the earlier, the sooner the stimulus of necessity is withdrawn or suppressed. Besides all this, there is a climacteric period in man as well as in woman. In woman it occurs soon after forty, or at the latest at fifty; but in man it varies between his thirty-fifth and sixty-fifth years. When it takes place in man, his character and figure both undergo a change, sometimes for the better, but sometimes for the worse. He becomes fat or thin, attenuated or obese. Old age sets in apace. The hair turns grey or white, the affections congeal, virility ceases; or, on the other hand, the figure remains lean and lank, the features are shrivelled, the hair falls off, and the complexion tans, while the mind improves, the wit sparkles, the understanding solidifies, and the flash of genius burns brighter than ever. The experience of a whole life comes into play; and the tardy seedlings of spring embrown the autumn of our days with fruit. In these cases, the organic life suffers at the cost of the cerebro-spinal system. But, on the contrary, we see the mind degenerate without our being able to account for it, in the most pitiable manner possible. Follies of the most deplorable kind are committed. The old man marries a young girl; and after having been respected for his frugality and prudence, suddenly breaks out and affects to play the boy, the gallant, and the fop. Sometimes, something worse than folly ensues. The religious man turns a worldling, the upright a spendthrift, the trustworthy a swindler; or he falls a dupe to religious enthusiasts and knaves, mistakes idealities for faith, fasts, prays, preaches, and insults the world.

No doubt, alteration of the brain is taking place *pari passu* with these alterations of character. It may be atrophy indicated by the loss of memory, slowness of speech and manner, and debility of gait and action. Or the circulation through the encephalon may be checked or impeded by ossification of the arteries, or softening of the coats of the cerebral arteries, or more distant disease about the heart and large vessels; or the neurine itself may be undergoing a change, particularly on its peripheral surface, as well as on the surfaces of its several ventricles or cavities. The convolutions become paler and the furrows shallower. The weight of the whole cerebrum and cerebellum is lighter, less complex, and seems to be reduced to the condition of the brain in early life. Softening of the surface of that delicate character which is detected only by letting a slender stream of water flow gently over it is sometimes the only discoverable alteration. But what is a very usual occurrence, and yet one that is often passed by unnoticed, because it is discernible only to a well-practised eye, which may not be present at the right moment for observing its attack, is a very slight fit of apoplexy and paralysis—so slight, indeed, that it occurs and passes away unperceived, and is recognised only in its after consequences and permanent effects. This appears to us to have been the case in Moore and Rogers; we have witnessed it more than once in private practice, and though loss of life does not ensue from it immediately, yet its ultimate effects are sooner or later fatal, and from the moment of its infliction, the patient is an altered being—he never recovers himself, but continues to exist, like a venerable ruin, with the marks of decay indelibly imprinted on his front.

Dean Swift used to say, there is no such thing as a fine old man, for if his head and heart had been worth anything, they would have worn him out long ago. This was the case with the late Sir William Hamilton, Bart., Professor of Logic and Metaphysics in the University of Edinburgh. He was an immense student; his head was a compendium of knowledge; he did not belong to the present world, but he was a living fossil of the age of Aristotle and Plato; the schoolmen of the middle ages, the writers of tough German, ideal Italians, and erudite Frenchmen. He spoke the language of another world, and nobody scarcely ever supposed he belonged to this. He was at work early and late, when he was young and when he was old, at meal time and at play; his mind knew no rest; and the consequence was that he was paralysed in the midst of his lucubrations and literary labours. His lamp went out, and darkness closed upon him before he could justly be said to be old. This was apparently a case of apoplexy with sanguineous extravasation upon or within the brain; and, perhaps, some softening besides. We speak under correction, as we are not acquainted with the results of the post-mortem examination, if, indeed, there were any autopsy at all.

He might have laboured less with his head, and in this way have prolonged his life; only had he done less than he did, he would not have been *the* Sir William Hamilton of Edinburgh. There is a very good account of his life in *The Leader* of May the 10th, from which we quote.

"We have to record this week the death of a man who, in the purely intellectual order of greatness, has hardly left his exact parallel in Britain, or even in Europe—Sir William Hamilton, Bart., Professor of Logic and Metaphysics in the University of Edinburgh. Born in Glasgow about the year 1790, and educated first in Scotland, and afterwards at Oxford, Sir William, who derived his baronetcy, with little or nothing in the shape of hereditary property attached to it, from ancestors of some distinction in Scottish history during the Covenanting times, adopted the Scottish Bar as his profession. He was called to the bar in 1813. Already at that time he had an extraordinary reputation among those who knew him, as a man of erudition and of speculative research. Younger men then living in Edinburgh as students, used to look up with veneration as they passed his house at night, to the lighted window of the room where they knew him to be busy with his books. His readings were of a kind at which ordinary men stand aghast—Aristotle and Plato; the Schoolmen of the middle ages; all German, all Italian, all French, all English, all Scottish philosophers. He was preparing himself to be a new name and a new influence in purely speculative philosophy—a man who, resuming in himself all that his predecessors in the series of Scottish metaphysicians had done, and bringing to the work of philosophy a culture, an acquaintance with universal literature, such as none of them had possessed, and perhaps also greater energy of nature, should again, in a utilitarian age, reinstate the old problems which Aristotle and Plato and the Schoolmen meditated, and call on the intellect of modern Britain to refresh itself by entertaining them, even if their solution was impossible. At length he obtained a position suitable to his genius and tastes. After holding for some time the chair of Universal History in the University of Edinburgh, he was appointed, in 1836, to the chair of Logic and Metaphysics in the same University. For twenty years in this position, he was an intellectual power, influencing sixty or eighty youths annually—teaching them a Logic, compared with which that of Whately is child's play, and a Metaphysics as hard and profound as that of Kant and his Germans, and yet clear-grained, genuine, and British. The admiration he excited among the students competent to follow him was unbounded, and none left his class without bearing his intellectual mark. It was always regretted by his admirers that his own insatiable passion for reading prevented him from putting forth works which would have conveyed to the world at large an adequate impression of his powers as a thinker. Even now, that he has left behind him is but a fragment of what he might have done. About the year 1829 he began to contribute to the *Edinburgh Review;* and the papers on speculative topics which he contributed to that periodical were, for some time, his sole literary manifestations of any importance. Scattered as they were, and fragmentary as they were, their influence on contemporary and subsequent thought was great; they were reprinted in France, as recognitions of a new philosophy; and in Oxford they helped to determine rising minds to new and more profound forms of logical and metaphysical studies. Some years ago, Sir William put forth an edition of Reid's works, with notes and dissertations, in which he expounded, by way of supplement to Reid, some of the cardinal notions of his own more advanced mental science. The book is one of the most amorphous ever issued from the

British press: it is very thick, it is printed in double columns in small type, and, what is worse, it is not finished, but ends abruptly in the middle of a sentence. And yet it is a book among ten thousand. In 1852 the articles in the *Edinburgh Review* were republished collectively, under the title of *Discussions on Philosophy and Literature*—a book as remarkable and better known. Before the publication of the *Discussions*, and, if we remember aright, before that of Reid, Sir William was seized with paralysis, which affected one side of his body, and to some extent also his speech. It was a sad sight to see such a man—a man, too, of fine physical appearance—moving about, thus crippled. His intellect, however, was unaffected by the shock; and he continued to the last, with some assistance, to conduct his class regularly every winter. Latterly he was engaged on an edition of the works of Dugald Stewart, which, we believe, he has left complete. He had an affection for this kind of work, which, seeing that it interfered with original labours, must be regarded as unfortunate. One is glad to know, however, that he has left his "Lectures on Logic and Metaphysics" fairly written out. When these are published, they will perhaps be the most perfect revelation of the man, in both his aspects—that of his colossal memory and acquaintance with the whole history of Opinion, and that of his native vigour and subtlety of speculative thought. It was the union of vast erudition with vast intellectual strength in pure speculation that made Sir William almost unique among his British contemporaries; and it is solemnizing to think that in one brief day such a brain may cease its thinkings, and such a memory, with all that lay gathered up in it, may be extinguished from the earth."

The imbecility of age is not so painful to the old as it is to those who stand by and wait upon it. With the return of our second childhood, we lose the consciousness of our prime. The loss of any of our senses is accompanied with the oblivion of its enjoyment. Thus, the blind are cheerful, the deaf happy, and the old content. So that we are tempted to conclude, that those exquisite lines of Goethe, so ably rendered into English by their noble translator, express a poetic fiction rather than a reality:—

"Give me the active spring of gladness,
Of pleasure stretched almost to pain;
My hate, my love, in all their madness,—
Give me my youth again!"

Although the sight of the angelic Margaret, as

"She sat by the casement's chequer'd glass,
The clouds fly by, and she watches them pass
Over the city wall,——"

meditating on her love, were sufficient to enkindle a spark of passion even in the icy veins of an old dotard. But no: in the really old, the flame is extinct, the ashes have been burnt out, and no spark can ever fire them again. We knew an aged gentleman, who, during the stunning effects of an apoplectic seizure, lost all his money by the failure of a bank. On recovering his senses, he could never be awakened to the feeling of poverty, nor the embarrassing consciousness of being a poor dependent on the bounty of his friends. Another gentleman, during a fit of apoplexy and its tedious consequences, lost two of his dearest relatives by death, and came into possession of some considerable property. On his recovery, he neither regretted the deaths, nor rejoiced at his own good fortune.

So delicate is the fine tracery of the nervous structure, that the damage of a single fibre or a set of fibres destroys the unity of the whole. It is like a grand orchestra, in which one instrument alone out of time or tune disturbs the harmony of the rest, and the finest musical composition in the world is entirely spoilt by its discord. And this serious evil is apparent, not only in old age, but even in the young, in whom the disastrous consequences of injury to the brain, &c. are far more important both to themselves and to the world. The following incident is a case in point:—

"A singular case of suicide, by a boy, was the subject of investigation before a coroner's jury, on Monday evening, at the Basin Tavern, Blast-lane. The deceased was a boy twelve years of age, the son of a respectable artisan in Tomcross-lane. It appeared that, on Wednesday, the 12th of March, Mr. Beardshaw had sent the boy on three occasions to Mr. H Unwin's steel warehouse, with a message respecting some steel, required in his trade of a table-blade forger. The boy neglected to perform his errand properly, and Mr. Beardshaw struck him a slight blow on the face, with his open hand. Two hours afterwards, the boy left the house, and was never seen again until Sunday, when his lifeless body was taken from the canal, by a man named Frederick Sale. He had not said anything when he left the house, and it was supposed he had only gone to some neighbours. When bed-time arrived, however, without his return, his parents became very uneasy, and the father, along with a friend, spent the greater part of the night in searching the town, but heard no tidings of him. To add to the anguish of the parents, there was discovered, amongst some old papers on the mantel-piece, on the third day of his absence, a slip of paper, containing the following, written in Roman capitals:—

'Art thou gone? shall thy step on the green hills no more
Give the echoes of music that charmed us before?'

Beneath this couplet the deceased had written—

'I am going to drown myself, so that you must go to the canal, and you will find me there;—it is for you hitting me. GEORGE HERBERT BEARDSHAW.

On the finding of this note, the father applied at the Police-Office, and consulted some friends, all of whom concurred in thinking that the boy could not have carried out the threat contained in this letter. They were led to this conclusion from the circumstance related by the father, that the boy had, in February, when sent an errand with 4s. 6d., absconded, and walked all the way to Liverpool, where he was detained by some friends of his father, and sent back to Sheffield. The assumption of his not having carried out the threat contained in his letter was further confirmed by the information that a boy, answering precisely to his description, was ascertained to have been sleeping one night at the coke-ovens. The father, although he could not bring himself to believe that his son had drowned himself, yet (as he stated to the jury), 'walked up and down the side of the canal night and day.' After nineteen days of fearful suspense, the sad news reached the parents, on Sunday, that the body of their son had been found floating in the canal, under one of the arches of the railway. The body presented the appearance of having been in the water nine or ten days. The somewhat vague information, therefore, of the boy leading a vagabond life for about a week after he absented himself from home, was very probably the truth. It was manifest, from the note left by the boy, that he was aggrieved by the chastisement inflicted by his father. The enquiries of the coroner were, therefore, particularly directed to the treatment the boy had received at the hands of his parents, and the information on this point was most satisfactory. All the evidence went to show that their treatment of him had been uniformly kind and indulgent. The blow of the 12th of March, slight as it was, was the only punishment the boy had received from his father for the last five years. About eighteen months ago, the boy received an injury on the head by a severe fall from a dray, and subsequently, but more especially after his return from Liverpool, there was a marked change in his manner and disposition, and several circumstances were now related which went to show the great probability that the fall had injured his brain. The jury returned a verdict of found drowned."

The concluding paragraph gives the true solution of the story—he had received a *mechanical injury of the head, and morbid changes had been the result, affecting the moral will.* In a boy, no one doubts this explanation of the case, but in the old, the changes within the encephalon are equally as certain, although we are inclined to put down to old age what is due to the morbid changes incidental to advanced life. Had not the mind been already sapped by organic alterations, proceeding at a slow pace, the reverse of fortune, usually made use of for explaining the mental malady, would not alone have been sufficient either to account for or cause the catastrophe. In cases of suicide, whether in the old or the young, the mind is generally affected: some imaginary evil—imaginary, because morbid—is the instigating motive, magnified to the last degree by a mind already irritated by the constant inroad of disease, and the consciousness of a calamity which is impending, in fact, from within instead of from without the body. And unhappily, too, the propensity to suicide is an hereditary one, because the predisposition leading to it is born with us, grows with our growth and strengthens with our strength.

The delusions to which a disordered condition of the sensorium is subject, are as various as they are calamitous, as the following fact illustrates:—

"A German, an insane man, was arrested on one of our wharves, by Police-officer Boardman. His insanity consisted in his having purchased a dory for $4,50, which he had fitted out in a peculiar manner, with oars and sails, and provisions sufficient only for a fortnight's subsistence. He had covered the boat over with canvas, except one spot, which he had left open in order to admit his person. It was his intention to put to sea in a day or two in this boat, hoping, as he expressed himself to Dr. Stedman, who was called to examine him, to reach Europe in twenty-two days. On the doctor's asking him how he should supply himself with food when what he now had was consumed, he said he had a little money to buy more. Whether he had the notion that a half-way house was to be found on the great deep does not appear. His insanity would seem to have arisen from home-sickness—not an uncommon cause of mental malady among our emigrants."—*Boston* (U.S.) *Traveller.*

We have no doubt that a careful dissection of the brain and spinal cord, would, in this case, should the opportunity ever present itself, reveal some very interesting particulars in morbid anatomy. The disregard of self-preservation allies itself closely to suicide, while it exhibits in a very striking point of view the serious calculation on wrong data so often betrayed by the insane, in the various modifications of dementia. There is a sober disregard of self and consequences, in the above case, that is perfectly terrific, except to those acquainted with the inmates of the madhouse, where such things are looked upon as the sign conclusive of a lost mind.

Nor is this all that can be said of the strange dreams of madness. The subjoined case is singularly illustrative of the fact that murder may be committed by persons whilst in a state of semi-consciousness, or in that confused and dreamy state of mind which often exists between sleeping and waking :—

"A correspondent of the *Richmond* (Va.) *Despatch* (U.S.), writing from Carolina, states that a few nights since Mr. William M. Kelley, of that county, was suddenly aroused from his sleep, and under the impression that his house was being broken into, seized his gun, and instantaneously fired upon some one, as he thought, entering the door; but, to his horror, he found that he had shot his wife, who was fastening it. The shot entered just in front and above the right hip, penetrating deep into the body. Two physicians were immediately called in, but found her beyond hopes. She lingered resignedly and uncomplainingly until about four o'clock on Saturday morning, when she died, leaving an almost distracted husband, an infant son eleven months old, and a large number of relatives and connexions to mourn her loss."

Dr. Pagan refers to the following interesting case (quoted by the Editor in his letter to the *Times*, referring to the mysterious deaths of Dr. H. Franck and his son at Brighton) to prove that murder may be committed by a person under the effects of a frightful vision :—Bernard Schedmaizig suddenly awoke at midnight. At the moment he saw a frightful phantom, or what his imagination represented as such—a fearful spectre. He twice called out, "Who is that?" and receiving no answer, and imagining that the phantom was advancing upon him, and having altogether lost his self-possession, he raised a hatchet that was beside him, and attacked the spectre; and it was found, alas! that he had murdered his wife.

The thirst for murder so peculiar to *maniacs* is pourtrayed in the following horrid narrative :—

The *Newhaven* (*U.S.*) *Journal* gives full particulars of a most frightful case of double murder at Woodbridge (Connecticut). It appears that a crack-brained fellow named Sanford (who had been several times in a lunatic asylum), waylaid a gentleman named Sperry, as he was travelling in a sleigh, near a gloomy piece of wood, contiguous to a cross-road, and murdered him with an axe. It would seem that Sanford with the head of the axe struck Mr. Sperry on the temple, inflicting a severe wound about three inches long in a horizontal direction over the right eye. It is supposed that this felled him to the ground beside his sleigh. Sanford then struck him again upon the back of the head, cutting a fearful gash behind the ear. As he rolled in the gutter upon the north side of the road, Sanford proceeded to cut his throat, which was evidently done with his axe. While the murderer was thus despatching his victim, the horse belonging to Mr. Sperry quietly walked on towards the turnpike. The murderer then went on to the house of Mr. Ichabod Umberfield. He went into the kitchen, and there found Mrs. Deming, a woman that does the housework for Mr. Umberfield, washing the floor. He put his arm around her waist, and told her he wanted her to go into the entry or hall, where he had deposited his axe and a hickory club when he came into the house. She slapped him across his face with her hand, and immediately left the room. He then went into the entry, and took the axe and club and passed into the other room, placed them upon the floor, and sat down by the stove. A little girl in the room ran into the bedroom, and lifting up the sash called out to Mr. Umberfield,—"Charles Sanford is in the house with an axe, and he is crazy—you must come in." Mr. Umberfield came into the house immediately, and sat down by the stove. He spoke to Sanford, asked him how he was, &c. Sanford made no reply to Mr. Umberfield, but sat in a sullen mood, apparently for about two minutes, and then arose and took up his axe and club (as the people in the room supposed) to go away. He passed behind Mr. Umberfield, towards the door, then suddenly turned around, lifted his axe and struck him a powerful blow upon the head. Mr. Umberfield fell to the floor with a groan, when Sanford struck another blow, and then deliberately cut his throat with the axe, nearly severing his head from his body. Only about four inches of skin upon the front part of his throat, and his windpipe, were all that connected his head with his body. The first blow probably killed him, as it fractured his skull extensively. The blood flowed freely upon the floor, and the room looked like the den of a murderer. The little girl screamed and ran out of the room, and he followed her across the floor, saying, "Stop your noise, or you'll all get your heads chopped off." Mrs. Deming opened the door just in time to see the murderer strike the last blow. Sanford went out of the house to wipe off the blood from his axe upon the snow, and while he was doing so the inmates of the house fastened the doors, and prevented his coming back again. He then left the house, passed out into the road, and following it but a short distance, soon struck off into the woods at the foot of West Rock. The ruffian was pursued, and captured after a desperate effort. One of the party who was armed with a pitchfork thrust it into Sanford's chest, and held the maniac at bay for a moment, until another struck him with a club and knocked him down. There were eight men in the party, and it was with the greatest difficulty that they could secure and bind him. He fought desperately. They finally got him secured, and took him to the house of Mr. Umberfield, and thence he was taken to gaol by officer Doolittle. He said he turned to go back to kill the whole of Mr. Umberfield's family, and would probably have done so if he had not been immediately arrested. He said, on his way to gaol, that he killed Mr. Sperry because he "had a cramp; and he killed the man to prevent the cramp killing him." He talked incoherently all the time, and when he arrived at the gaol presented the appearance of a raving maniac.—*Times*, 22nd Jan., 1856.

This story reminds us of the wholesale murders that happen every now and then among the semi-barbarous people of the east, such as the Malays and Chinese of Cochin China, for instance. A wretch is seized with a sudden fury for bloodshed. He draws his sharp knife from his side, kills the first person he meets with, and then proceeds at a furious pace, striking at random and wounding or killing all that come within his reach, till he himself is at last knocked down and despatched, like a rabid animal. As many as sixty desperadoes of this kind have been known to band themselves together, armed with their knives, and sallying forth on their errand of blood, to go on slaughtering and wounding all around them, till they themselves are at length slaughtered in return. So fierce has their determination been at times, that they have repulsed a detachment of regular troops sent against them on purpose to put them down. In Europe we may safely declare we know nothing of horrors such as these, which seem to belong to the heathen populations of the world, and those portions of mankind that lie external to the pale of Christian civilization. It is not attributing too much to ourselves to suppose that our high moral discipline is owing to our stricter and more religious mode of education; only do not let us congratulate ourselves on our own proficiency in moral worth too soon; we may not shed blood so freely (always excepting the passion for legitimate warfare, which is called *glory*) as the darker races of mankind, but we have not advanced much beyond the dark portals of heathenism, if, as seems to be the case, the purport of the subjoined letter to the *Times* relative to the traffic in women is unquestionable:—

"In a leading article of *The Times* (Thursday, March 20) you have commented with just horror and indignation on the infamous traffic in young girls, at this time carried on to a greater extent than can be conceived or believed by those who sit at home, intrenched round by all the sanctities of domestic life and all the safeguards of virtue. In the course of the judicial inquiry which gave rise to your remarks, it was stated publicly that this traffic has become a 'system,' and a source of profit; that the law cannot reach it; and that without the intervention of our Foreign Minister, it is not likely to be put down.

"That such an infamous traffic does exist has long been well known to me and to others. Not only is it true that English girls are inveigled out of this country in such numbers that, as I remember, an association was formed in Paris to protect them; but it is not less true that for the same horrible purpose girls are brought over to England from France, from Belgium, from Germany; it is, in fact, a trade under all the conditions of export and import —a trade which, if not legalized, is tolerated; and I have myself heard it, I will not say defended, but accounted for, excused, as the necessary, inevitable result of certain permitted social vices. When several trials relative to these foreign victims were reported two or three years ago, and sent a strong shudder of horror and disgust through our virtuous society, *The Times* was blamed by some persons for the publicity given to the circumstances and the severity of its comments; but others who recoiled from such details felt wisely grateful for the exposure of such unmanly vice, and for the manly scorn and detestation with which it was visited.

"In this recent case, not women only, but all right-minded and generous men, have reason to thank you for the part you have taken. You conclude your denunciation by an appeal to Englishwomen, and (printing the word in capitals to enforce your appeal) you require that Englishwomen should 'lay to heart'

such a state of things, and use their utmost power to stop the progress of this enormous wrong.

"I am an Englishwoman, and, in common with many other Englishwomen, feel the shame and horror of such a state of things; but will you, who thus appeal to us, or will any of your correspondents, point out what it is our duty to do?—how we are expected to act, to speak, or even to think on such subjects? We have been told heretofore by men whom we respect, that it becomes women to be absolutely silent on such revolting topics—to ignore, or rather to affect to ignore, such a 'state of things' as you allude to. We have been told that, in virtuous women, it is a breach of feminine delicacy even to suppose the existence of certain outcasts of our own sex, or of certain exemptions in regard to vicious indulgence assumed by yours; in short, that, as women of virtue, we have nothing to do with such questions, though we know, too well, how deeply they affect us, how terribly near they approach us personally, how the far-reaching contagion of such covert vice involves in some form or other the peace of our 'virtuous' homes, the fidelity of our husbands, the health and morality of our sons, the innocence of our daughters. We have been allowed, indeed, to patronize penitentiaries, to read chapters of the Bible, and distribute lugubrious tracts to wretched, sullen, disordered victims; but, meantime, we are told—I have myself been told, half pityingly, half sneeringly—that for every one unhappy creature we rescue out of the streets, two will be at once supplied to fill up the vacancy; that this 'state of things' is a necessary social evil; and that we virtuous women had better not meddle with it, lest worse befall us.

"So it has been said in former times; but it seems, from the appeal you make to us, that in these days Englishwomen may feel, may think, may speak out on such subjects; may, without reproach, take such a part in their discussion as becomes the members of a Christian and civilized community. But what are we to do, where law is weak, where custom is strong, where opinion is cowardly or wavering, where our very knowledge involves an imputation on our feminine decorum—what are we to do? A popular journal, in reference to this trial, intimated that where the law cannot reach them it is permitted to take the chastisement of such vile panders and procuresses into our own hands. Does this mean that they should be pilloried or pummelled to death in our public streets? I believe this would be their fate if they were once recognised, but where would be the justice of it? Shall we stone those who minister to vice, and spare those who practise it? That class of wretches whose sole and profitable occupation it is to hunt down and ensnare victims becomes, we are told, more and more numerous, more and more audacious; but for whom are the victims hunted down and ensnared, imported and exported as so much merchandise? So long as the market exists, the article will be supplied—tell us, therefore, what are we to do? The education of your sons does not rest with us. In the schools where boys are collected together, generally far out of the reach of pure, healthy female society and influence, the first thing they learn is to despise girls; and the second, to regard the impetticoated half of the human species as destined for their service or their pleasure; hence in the higher and better educated classes early impressions which lead to the most selfish and cruel mistakes in regard to the true position of women, and in the lower, more ignorant classes, to the most terrible tyranny and brutality. Against the latter, it is said, our Legislature is preparing stringent measures, but against the former what is to defend us? I speak in the name of Englishwomen to whom you have appealed, and ask counsel and help from generous and thoughtful men—what are we to do?"

That it should be possible to tell such a tale as this in the present age, is incredible; but it is still more incredible, that the whole world should listen to its recital without rising up unanimously to put a stop to it, in a just fit of shame and indignation! Is it that we are lending ourselves to its existence? or, is it a crying evil beyond redress? Had it been but hinted at by Tacitus of Rome, or scoffed at in Athens by Aristophanes, we should have rushed forward eagerly to join issue with the comedian and historian against the gross corruptions of *their* times. What a fruitful theme it would have proved to every exalted moralist and acute politician who aimed at present popularity, by pointing out the vices of antiquity and the virtues of the moderns. And yet, here it is upon the very thresholds of our dwellings—lying at our feet—walking the public thoroughfares at noonday—living amongst us—travelling in the same railways and crossing the Channel in the same passage boats—sitting at the same board in the same cabin, or at the same *table d'hôte* with ourselves, our wives and daughters, and yet there is no one with sufficient moral courage to rise up and denounce the loathsome monster and scout it from society! O, shame upon modern refinement and virtue! O blot and blemish upon the wealthiest and most prudent of the nations calling themselves civilized! Come forth, thou reprobate pagan of old,—rise up from your nether realms of Pluto's dreary reign, and stand amazed at the cold indifference of the first Christian community upon earth! Say where the root of the evil lies, and show where the first blow of the axe shall be aimed with precision and effect—what, where, and how? There! replies the spectre—there, *in the nursery and school*—there—strike home before ever the sapling has grown up into a tree, and spread its fatal branches far beyond your reach. Lop vice in the bud, or, as your own Wise Man tells you, Train up the child in the way it should go. Any other remedy is useless, continued the fading seer; you must teach morals as well as science, self-government as well as trade, religion as well as manners; or, remember, *consueta vitia ferimus, non reprehendimus.* He said, and vanished.

"One of the strangest of vexed questions is the question, 'Have Animals Souls?' To the majority of modern Christians, thinking and unthinking, it seems eminently absurd, if not eminently 'dangerous,' to maintain that animals have souls; although to ancient Christians, as well as to ancient philosophers, the absurdity would have been in the denial, *Anima*, from which the name is derived, meaning the breath of life, and ψυχη, meaning, as we have shown in these columns, life *and* soul, indifferently—for in truth the two were not separated until modern metaphysics, probably among the schoolmen, came to divorce them, and make them essentially independent.

"An able writer in 'Putnam's Monthly' for April, takes up the question. He first adduces Scriptural evidence of 'one and the same covenant binding us and animals to our Maker,' and justly remarks on the deplorable habit of using the word animal as a term of contempt. All contempt is perilous, but contempt of God's creatures in their free activity is essentially irreligious. Of plants, and even of stones, we speak with veneration and admiration, but the

'brutes that perish' we permit ourselves to vilify. Curiously enough, the nearer these brutes approach our own proud selves, the deeper is the loathing expressed for our 'poor relations,' as Luttrell wittily called monkeys; and many a worthy gentleman would drop your personal acquaintance if you suggested to him that the dog which loves and obeys him has a soul not essentially different from his own. The writer in 'Putnam' argues, and justly, for the inner life even of plants; which will be paradoxical only to the immature psychologist. His case is better made out with animals, however, because we are more acquainted with the functions of animals. Read this:—

'Animals discern their food, as the first condition of their existence. The tree, also, it is true, uses all that nature has placed within its reach for self-preservation, as if it were created solely for its own purposes; but it does so mechanically, constantly, and without choice. The animal, on the contrary, knows its food from afar, seizes it with all the eagerness of instinct, and disposes of it in the most useful manner. In order to distinguish food, it must have been placed by the Creator in a pre-established harmony with its food; it must have apertures to seize it; and a space within to hold it. These, however, are not given to all; for some, that dwell in the water, are mere ribbons or threads, balls or cylinders. How they absorb, we know not. The infusoria, however, have each a stomach and often several; they even begin to fight for their food. Others are endowed with cilia—tiny hairs, that whirl in restless motion around the mouth, and fill it with invisible victims. How different from the grim medusa, that sends out eighty thousand arms, a whole army, eager with insatiable hunger. The shark swallows men, horses, and oiled powder casks; the wha e entire hosts of sea animals. Other cunning creatures are more fastidious than the most experienced gourmet. The silk-worm eats only mulberry leaves, and a suspicion of dampness deprives him of his appetite.

'There is a large wasp that lives in sand-burrows and indulges in eccentricities like few other beings: the only animal, save the horse, that sleeps standing, and so it dies. You see its lean, lank body, stand prim and prudish near its former dwelling—you touch it, and it falls into dust. It proudly refuses to lie down, like other poor insects, and decently to fold up its limbs. But its pride is still greater in its choice of food. It catches spiders, butterflies, and caterpillars; but, instead of killing them at once, it only bites them in the neck, paralyzes them, and drags them into its little hole. Who taught it to deprive large insects of wings and legs, and to leave the smaller unharmed? It rejects all alms and gifts. You may choose its choicest morsel and place it before the hungry wasp, it will not touch it; if you put it, during the owner's absence, into his house, he indignantly ejects it on his return.'

"Again:—

'The cunning ants keep cows in their stables. Almost every anthill, belonging to one variety, has a beetle in it, who lives, rears a family, and dies among them a welcome and honoured companion. When the ants meet him they stroke and caress him with their antennæ; in return he offers them a sweet liquid that oozes out under his wings, and of which the little topers are passionately fond. So great is their attachment to the odd confectioner, that they seize him, in times of danger, and carry him off to a place of safety; the conquerors of an invaded nation spare the sweet beetle, and, what is perhaps more surprising, his maggot and his chrysalis, though themselves utterly useless, are as safe among their wise hosts as if they also possessed the luscious honey. Other ants, again, keep countless aphides, that sit on the tender green leaves of juicy plants, as on green meadows, and suck away so lustily that their delicate little bodies swell like the udders of cows on rich spring pasture. At that season, the ants have to feed their young with more delicate food than their own; they stroke and caress their tiny milch cows, gather the nutritious liquid that pours forth under their sagacious treatment, and carry it, drop by drop, to their nurseries.'

"All this, we know, is called Instinct, and much of it is probably not more psychial, in the usual sense, than the union of an acid with a base. But the human soul is also mainly composed of Instincts, although these are less obvious owing to the complexity of higher psychical operations. It is evident that the simpler organisms will manifest simpler instincts and activities than the more complex organisms; the philosopher's business is to identify the 'unity of composition' in the psychial as in the anatomical world, and to show that animals only differ *inter se*, by differences of degree.

"Besides the simplest of all instincts, that of discerning food, there are others also very simple, and consequently universal—the discernment of a proper domicile, or habitat, for example. The essayist has enumerated some curious facts on this point. He allows his imagination to run away with him occasionally in speaking of the instinct of self-preservation; and when he says that the 'cunning beetle feigns death *because* crows do not touch dead beetles,' he is talking the loose talk of Natural Theology, not science. In the same way, when discussing whether all animals feel the sensations of hunger and thirst, he outruns observation and allows imagination to interpret. 'Grasshoppers are the first creatures that are known to satisfy thirst by drinking.' How is this known? 'They are passionately fond of sipping the dew of the morning.' That they sip the dew is a fact of observation; but no observation, no gleam of evidence reveals that they do so with 'passionate fondness.' Generally animals which live on liquid food do not drink; whilst birds which eat dry seeds are ever thirsty. 'Hence it has been often asked, why drinking and singing should ever be found so closely bound to each other?' A question for hilarious gentlmen who over a 'social glass' are prone to indulge in bursts of lyrism, and who alternately 'pass the rosy' and *toll de roll!*" — *The Leader.*

The error in the foregoing paragraphs consists in not drawing the distinction between the words *soul* and *understanding.* To possess a soul, implies moral responsibility; but no one ever dreamt of imputing moral responsibility to any animated being lower than man; so that no animal can, in the strict meaning of the word, be said to possess a *soul.* It seems idle almost to debate the question. It has never been seriously mooted except among obscure and depraved enthusiasts, such as the ancient Brahmins, the Manichees of the first ages, and Pantheists of the lowest description. But *understanding* is possessed by all animated beings in various proportions and degrees: the sagacity of the dog, the sharpness of the ape, and the intelligence of the elephant, are proverbial. Even *moral affections* are enjoyed by animals and insects: the horse is docile, the lion courageous, the lamb timid, the spider deceitful. All these qualities are fragmental portions of the understanding distributed severally throughout the creation for the particular use and purpose of each being; and are, when taken together and summed up into one, the human understanding complete. And, were not the various qualities of the understanding manifested by animals identical with that possessed by man, there could be no intercommunion between man and animals, for without this mutual intelligence, the rider could not manage his horse, nor the sportsman direct his dog, nor even the pig-boy drive his pigs. Animals and man must understand each other, otherwise animated nature would be a confusion. Even sounds of the voice and the meaning of words are understood by animals as distinctly and fully as they are by ourselves; and the intent and object of our actions are perceived by them in the same sense as we intend them to be perceived. Thus the horse knows the sound of the trumpet, the smack of the whip, and the driver's bidding; the hound responds to the huntsman's horn, the cat minds the maidservant's call, and the cow knows the cry that drives her home to be milked. Stories are told of serpents that have become familiar with man, of insects that have mated with the prisoner in his cell, and of hares that have sat like cats before the fire. The mechanism of the beaver is like our own, because ours is the same as his; and the fox pilfers our yards with the same adroitness as the thief pilfers our coffers. Thus the intelligence of animals is the comparative anatomy of the understanding of man: what is one in us is several in them. They are the analysis of the mind of which we are the standard and type. By pursuing this train of reasoning, we might show that the less perfect understandings in man approximate to the lower understandings of animals. Thus we say, as stupid as an ass, as filthy as a swine, as timid as a lamb, as cruel as a tiger. The higher human understandings admit not of any such debasing comparison, since they cannot be likened to anything below themselves. Great minds are not brutal, but, on the contrary, so elevated, that they cannot be lowered by any comparison. They are THEMSELVES both in understanding and soul, and comprehend within themselves all the mental qualities of every animated being below them. It is this pre-excellence that can never be predicated of any of the inferior animals, and entirely excludes them from the idea of possessing either a soul or an understanding, in the fullest meaning of the words.

Virgil, indeed, has placed his horses with his heroes in the Elysian fields, and the horses of heaven are mentioned in Scripture, both in the Book of Revelations and when Elijah was carried to heaven. But we apprehend that no reader ever regarded these relations in any other light than that of sacred or poetic imagery of the loftiest kind; and every one would be extremely shocked were he called upon to suppose that animals will be called to judgment at the last day along with the rest of the human species.

Whether every crime of the same kind is equally deserving of the same punishment is a question that has not yet been reduced to a practical solution. In the eye of the law, wilful murder is regarded only in one light; but in a moral point of view it is capable of receiving many degrees of comparison. The following observations bear upon the matter:

"The execution of Elizabeth Brown is a questionable matter, following as it so soon does the extension of mercy to the wretch Celestina Sommers. Brown killed her husband, according to her own confession, as follows:—'My husband, John Anthony Brown, deceased, came home on Sunday morning, the 6th of July, at two o'clock, in liquor, and was sick. He had no hat on. I asked him what he had done with his hat. He abused me, and said, 'What is it to you, —— you?' He then asked for some cold tea. I said that I had none, but would make some warm. He replied, 'Drink that yourself, and be ——.' I then said, 'What makes you so cross? Have you been at Mary Davis's?' He then kicked out the bottom of the chair upon which I had been sitting. We continued quarrelling until three o'clock, when he struck me a severe blow on the side of my head, which confused me so much that I was obliged to sit down. Supper was on the table, and he said, 'Eat it yourself, and be ———.' At the same time he reached down from the mantelpiece a heavy horsewhip with a plain end, and struck me across the shoulders with it three times. Each time I screamed out. I said, 'If you strike me again, I will cry 'Murder.' He retorted, 'If you do, I will knock your brains out through the window.' He also added, 'I hope I shall find you dead in the morning.' He then kicked me on the left side, which caused me much pain, and he immediately stooped down to untie his boots. I was much enraged, and in an ungovernable passion, on being so abused and struck, I directly seized a hatchet which was lying close to where I sat, and which I had been using to break coal with to keep up the fire and keep his supper warm, and with it (the hatchet) I struck him several violent blows on the head, I could not say how many. He fell at the first blow on his head, with his face towards the fireplace. He never spoke or moved afterwards. As soon as I had done it I wished I had not, and would have given the world not to have done it. I had never struck him before after all his ill-treatment; but when he hit me so hard at this time, I was almost out of my senses, and hardly knew what I was doing.'

"If this account be true, the murder was the result of great provocation, or rather the crime was not murder, but manslaughter; for there was no deliberation—the woman, in her passion, having taken the first weapon that came to hand to protect herself and to disable her brutal assailant, who might otherwise have killed her. But the convict's own account of what occurred is, of course, open to suspicion. True. It is, however, to be observed in favour of the truth of the confession, that it was made immediately before the woman's execution, and when no hope could have been entertained that the sentence of the law could be stayed by it. Brown's conduct, too, was some pledge for sincerity. She behaved with great propriety in prison, and met her fate with calmness and fortitude.

"Assume, however, that the confession is valueless, and then all that is known of the murder is that a jealous wife killed her husband; but whether with circumstances of provocation or not, cannot be affirmed on the one hand or denied on the other. It is only certain that the husband was drunk—a fact which raises reasonable presumptions against his conduct, and that he was of profligate habits which may have provoked the natural resentment of his wife.

"Now, compare this case with that of the favoured Celestina Sommers. Whether the extenuating circumstances stated by Brown be true or not, there is not on the other side a particle of evidence showing any cruelty beyond that which necessarily belongs to the destruction of human life. In the other instance—that of Sommers—more than the fact that she had killed her child was known. It had been no hasty deed. The wretch had deliberately inveigled the poor child into her den, had taken her into a cellar, and then proceeded deliberately to cut her throat, replying to prayers for mercy with asseverations of her murderous purpose. It is certain that in this atrocious case there were all aggravating circumstances, amounting to a degree of wickedness almost unparalleled. It is possible, nay, probable, that in Elizabeth Brown's case there were extenuating circumstances; and it is certain that there were no known aggravating circumstances. Yet Brown has suffered death, and Sommers has had the benefit of the Crown's clemency. Strong representations in favour of Brown were not wanting; but Sir George Grey did not find in her case any of the circumstances that moved him to extend the mercy of the Crown to the merciless Celestina Sommers. Inscrutable, unaccountable, and utterly inconceivable, are the reasons of the Home Office for rigour and indulgence."—*The Examiner.*

But another question arises out of this, namely, as to the moral worth of public executions. We copy the following pertinent remarks on the subject from a contemporary: *

"It has long been felt, and often been repeated, that public executions do more harm than good. That the effect produced by them upon the crowds which they draw together is pernicious, can scarcely be denied. The levity exhibited by the populace which makes the distressing scene of a fellow-creature's death a holiday show, is sufficiently indicative of moral mischief. Few out of the thousands assembled to witness the awful spectacle, return from it with serious thoughts and solemnised feelings. All the rest go away, more callous, more depraved than they were before. Even the few persons of respectability, who may be numbered among the occupants of the windows commanding the scene, can hardly be the better for this gratification of a morbid curiosity. The rabble which meet beneath the gallows learn their lessons of brutality, of reckless indifference to human life; the criminal portion of it i rendered more daring under the unhealthy stimulus of having seen "the worst" that the law can do in the way of punishment.

"The evil of all this, which is recognised and admitted on all hands, has been very much aggravated, of late years, by the fact, that it has furnished the advocates of the abolition of capital punishment with one of their most plausible pleas. Unfortunately, it has become the fashion to talk of punishment as if its sole, or, at least, its principal object, was to serve as an example of warning, and to deter others from the commission of crime. The idea of "the vengeance of the law" is all but universally scouted,—thoughtlessly denounced by many who ought to know better, because they profess to reverence, and many of them doubtless do reverence, the authority on which that idea is founded. The recognition of that authority, the acknowledgment of the fact that the civil magistrate is a power ordained of God to bear the sword for the punishment of evildoers; and, as regards capital punishment, of the principle enunciated by the same authority, that 'whosoever sheddeth man's blood, by man shall his blood be shed,' is the best, rather it is the only and all-sufficient answer, to the various fallacies put forth by those who would expunge capital punishment from our penal code. But since there are many who cannot be brought to take this view of the question, who will not admit the lawfulness of capital punishment, or, indeed, of punishment of any kind, upon any other ground than that of deterring the criminally-disposed from the commission of crime, it is a pity that the circumstances under which the extreme punishment of death is inflicted should be such as to supply a plausible plea for its abrogation. And this the effect of public executions undoubtedly does.

"A new element, however, has now been imported into the question. It appears, from what took place in the Court of Common Council, that there is foundation for the report which attributed the trepidation exhibited by the hangman, at the execution on Monday se'nnight, to an anonymous letter which he had received, containing threats against his own life. Making every allowance for the part which the posture in which the wretched criminal was placed on the scaffold, in consequence of his real or simulated prostration, had in prolonging his sufferings, and affording him the opportunity of making those vain struggles for life of which it is impossible to think without a shudder, it is clear that the revolting spectacle which ensued would either not have taken place at all,

* "John Bull."

or would have been put an end to at once, if the executioner had been possessed of his usual nerve and presence of mind. That those to whom the execution of the law is committed,—and this consideration is not necessarily confined to the hangman, it may reach higher, for fanaticism is ever soaring,—should be exposed, in a necessarily defenceless position, to the murderous attempts of any madman that may mingle in the crowd, is not to be tolerated, in the interest either of justice or humanity towards those whom the law claims as its victims. The fact that a threat of this kind had been resorted to, for the purpose of deterring the hangman from the execution of his office, is a strong argument against the practice of public executions.

"In the face of such a fact, it is difficult to understand how an officer of State, placed in so high and responsible a position as that of the Home Secretary, could give such an off-hand answer on the question of considering the propriety of a change in the system which makes the execution of criminals a public exhibition, as that made by Sir George Grey to the inquiry addressed to him by Mr. Biggs. The matter will not, we trust, be allowed to rest there. The revolting spectacle of Monday se'nnight is one, the repetition of which cannot and must not be hazarded. The lesson conveyed by it ought not to be lost upon the Legislature. The remedy is simple and easy. There can be no difficulty in surrounding the execution of the extreme sentence of the law with such safeguards of supervision, on the part of the public, as shall effectually prevent the possibility of public justice being abused for purposes of legal murder, or desecrated by attendant circumstances of barbarity. Execution within the prison walls, under the eyes of spectators summoned to it as witnesses, who attend as a matter of painful duty, and not for the gratification of a morbid curiosity or a depraved taste for horrors, will not be less solemn, nor less effective in deterring from the commission of crime. It will have the additional advantage—not an unimportant one, considering the eternity in which the culprit is launched by the hand of the hangman—of concentrating the attention of the criminal about to pay the forfeit of his life, upon his own wretched condition; of removing from him all temptation to bravado; and of sparing him the distraction and disturbance which can hardly fail to be produced upon a man's mind by the sight of a multitude assembled to gaze upon his death as a public spectacle."

It has also been asked, why not abolish capital punishments altogether? to which the constant answer is, that some crimes can be restrained only by the death of the criminal. Of course, to the criminal himself, death, though a dreadful catastrophe to think of, is yet no penalty, humanly speaking. The criminal dies, and there is an end of him. The benefit of capital punishment, then, must remain for the good of the public, which is certainly benefited, if the knowledge that such and such crimes forfeit the life of the culprit shall be found sufficiently coercive to restrain the survivors from the perpetration of similar offences. Society is bound to protect itself; and the common consent of the world votes in favour of the punishment of death for certain trespasses against its life and property. But it by no means follows that what is called the *common consent of mankind* should be implicitly accepted as the unerring rule of truth and justice; for if so, let us return to the rack, the thumb-screws, the Lynch-law, or the bow-string, to which the world has before now given its common consent, as if such things were infallibly right. Perhaps they were proper for the time being; the Lynch-law in unsettled communities, and the bow-string in the days of despotism. But times alter, and things alter with them, and our judgment alters with the alteration of things and times. In settled states, many severities may be safely dispensed with; and as the Lynch-law gives way to the trial by jury, so the capital punishment by death may yield to the milder punishment of exclusion from the world for the rest of the culprit's life, or a portion of his life; and society, as it is now constituted, is surely strong enough to protect its own interests and safety, without being in haste to take away life from those who threaten to molest them, or have, in fact, already molested and damaged them. As to the popular cry of death for death, and the public feeling of satisfaction at the execution of a great criminal, we own we look upon such outbursts as nothing more than a hasty spirit of revenge, which had much better be suppressed than encouraged. To our ears, it has something of the sound of a savage yell in it. It would be better if the daily press were to endeavour to moderate such passionate expressions of abhorence for vice or crime than to join in the outcry and sympathise with its over-sensitive exclamations. Life is life; and because A has killed B, it is no reason why we should rush upon A and kill him in return. A might repent, or at all events we might allow him place for repentance; and, while protecting ourselves, afford him the opportunity of reflecting on the past and meditating on the future, without endangering our own security and well being by doing so. Such a proceeding on our parts seems more consentaneous with a people professing and calling themselves Christian, than an eagerness for putting in force the extreme severity of laws, which had their rise in days of barbarism and disorder that now no longer exist. It is with pleasure, therefore, that we turn to the account of the last hangman and the last gibbet.

"John Murdoch, who, until disabled by age and infirmity, about four years ago, had officiated for a lengthened period as the finisher of the law in Glasgow, and who was the last hangman who is ever likely to be in the ordinary pay of the corporation, has himself at length 'shuffled off this mortal coil.' For a period of above twenty years his stalwart form and grim visage (partially concealed by an old high-necked waterproof) were seen as the presiding genius at every scaffold which was erected throughout Scotland, and at not a few in the north of England. Murdoch, who was a baker, came to Glasgow from the north upwards of twenty years since, and was even then advanced in life. He was in poor circumstances, and contrived to get some humble employment about the corporation property. It happened that about this time Tam Young—the last functionary who had a formal appointment and a regular salary, and who wore the executioner's official costume—was getting rather shaky, and accordingly Murdoch was retained as a sort of assistant or stand-by. On Young's death he got a monopoly of the trade, such as it is; but, as he had neither the official appointment nor the regular pay, he was remunerated by the job. He took to the work quite genially, and, as he regarded his own functions as perfectly necessary to good government, he did not fail to be on perfectly comfortable terms with himself. As his person became known in Glasgow, however, he found it convenient for his comfort to remove from the city, and took up his residence sometimes in Paisley, sometimes in Kilmarnock, sometimes in the adjacent villages—such as Motherwell—and he has even been recognised officiating as a pastrybaker's assistant at one of our fashionable Clyde watering-places. The tidings of a murder case at a Glasgow circuit always drew him forth. As soon as the judges sat down he reported his presence to the authorities, and then waited patiently, in the hope that the man would be hanged. After sentence was pronounced he felt all right. That the mind of the magistrates might be kept perfectly easy as to no accident taking place at the eleventh hour,—for in this case, according to the old notion, the youngest baillie must do the work,—old Murdoch always lodged himself in prison a week or ten days before the event, where he had bed and board at the public expense, and thus he was certain to be forthcoming when needed on the morning of the execution. But, unfortunately for Murdoch, Glasgow alone did not afford employment enough to support a man of this trade when 'paid by the piece,' and accordingly he laid himself out as a peripatetic finisher of the law in general; and we suppose he referred to the magistrates of Glasgow for his character and qualifications. In this way it was his lot to string unfortunates up at Edinburgh, Inverness, Aberdeen, Ayr, Perth, Dundee, Stirling, and in various towns in the north of England, such as Newcastle, Carlisle, &c. Taking a pleasure in his work, he held anything in the shape of a reprieve or commutation in mortal detestation, and used to say that the ridiculous weakness of the Government did him out of the best job he was ever likely to have—viz., the hanging of Frost, Williams, and Jones, for which employment he was the favoured candidate. We have said he took a pleasure in his revolting work. He officiated, for instance, upon the two murderers Doolan and Redding, who slaughtered a railway ganger while the Edinburgh and Glasgow line was being constructed, sixteen years ago, and who were executed in a field close to the scene of the crime. As Murdoch stood at the bottom of the scaffold, immediately after the men had been thrown off, one of the authorities remarked that Doolan had not been properly handled, as he struggled and suffered much. 'It's his ain fau't,' said Murdoch, 'nocht wad ser' him but he wad tak' a jump when the drap gaed doon; but see, sir, hoo kindly Redding's slippin' awa' (dying). The last occasion on which Murdoch officiated in Glasgow, or anywhere else, we presume, was at the execution in October, 1851, of a man named Hare, for a murder committed in Blantyre. He was then eighty-four years of age, and was so lame with rheumatism that he had to hirple after the criminal to the gallows by the aid of a staff; but, once there, the old fellow did his duty with nerves of steel. Although there have been, unhappily, other occasions of the same kind since, the magistrates of Glasgow could not trust Murdoch again, and accordingly they called in the help of Calcraft, who, we trust, will be found perfectly able to do all the work of this kind which is required over the United Kingdom. Murdoch, being thus laid aside, went to the charming village of Bothwell, where, happily, his 'antecedents' were unknown, excepting to one or two, who generously kept the secret; and here he died, on Saturday, the 15th instant. He was maintained for the last few years by a monthly dole from the corporation of Glasgow, which was disguised, to the public, under the head of 'criminal expenditure,' and he will be the last official of the kind, we trust, who will permanently have a place on the chamberlain's books. He was not a native, and had no claim on Glasgow, excepting that which abject destitution gave him, and that he had been useful in performing necessary, but very vile functions. Had Glasgow left him to starve, it would not have been an easy matter to fix the parish liable for the support of a wandering hangman, approaching ninety years of age.

"A day or two ago the last gibbet erected in England was demolished by the workmen employed by the contractors making the extensive docks for the North-Eastern Railway Company upon Jarrow Stake, on the Tyne. The person who was gibbeted at that place was William Jobbing, a pitman, aged thirty years, convicted at the Durham Midsummer Assizes, of 1832, of being concerned with another pitman, James Armstrong (who absconded and was never apprehended), with murdering Mr. Nicholas Fairles, a magistrate, upon the road to Jarrow, on the 1st of June in that year, and was hung that month at Durham. In the summer of 1832 there was a lengthened strike of the pitmen of Northumberland and Durham. Bitter feelings arose between masters and men. Many hundreds of the families of the men were turned out of their cottages and lived in the lanes and roads in camps for months. The collieries had to be protected by military and an immense number of special constables, notwithstanding which three murders and many outrages were committed upon non-union men, and Mr. Fairles fell a victim to his zeal in endeavouring to maintain the law. At the time of Jobbing's trial an old law had been revived by the Whigs which condemned a murderer to the gibbet. Jobbing was the only person, we believe, gibbeted under that act; but so great were the horror and disgust of all parties with the sight of the body of the poor wretch dangling in chains by the side of a public road, that great gratitude was expressed when the pitmen took it down one dark night, and either sunk it in Shields Bar or buried it under the walls of Jarrow Monastery. It is, however, a gratifying fact, showing the progress of civilization among the mining population of those two counties, that though there have been several strikes among them since 1832, none of those strikes have been marked by a repetition of the fearful acts of violence of that year. At one of the great meetings of the pitmen, held in the spring of 1832, the late Marquis of Londonderry attended on horseback to remonstrate with them. But he had a company of soldiers with him, which were in hiding in the valley. This was known to the pitmen, and the pitman that held his horse's head as he spoke had a loaded pistol up his sleeve, in case the Marquis should wave the soldiers to come up, to blow the Marquis's brains out. Fortunately, the good feeling and kind heart of the late nobleman prevailed, and that emergency did not arise."

We can remember when the Kent side of the river Thames was decorated with many a gallows, upon which dangled the remains of culprits, being pecked at by the crows and aimed at by the boys. It was a sight held up *in terrorem* against criminals who had been convicted of murder, mutiny, &c. upon the high seas; and the common consent of mankind had agreed to the propriety of these horrid exhibitions. But now the same common consent has agreed to their removal. Not far from the spot where we are writing these lines is a pit called the *Dead Man's Hole.* A murderer had been hung there in chains to the terror of all future highwaymen: the highwaymen are no more, the gibbet and its loathsome remains have long since been removed. The common consent erected the gibbet, and the common consent has removed it. We can also remember when the criminal condemned to the gallows was drawn through the chief thoroughfares of London, stretched upon a platform laid across a cart, driven by a carman slowly through the gazing mob. What was the use of this display? The same common consent that approved of it has since then suppressed it. As better taste, the result of better feelings, springing from more experience, or from what is equivalent to experience, better understanding, gains ground, barbarities are seen to be nothing more than the provocatives of the very crimes they are meant to suppress. Society ought to show itself above the imputation of revenge, and to be able to stand firm and erect in the midst of these aggressions on its stability, which is too firmly grounded ever to be shaken by the exceptional assaults of wickedness, and too wise ever to allow itself to behave cruelly towards those whom it censures, convicts, and condemns.

We subjoin a curious extract from the "Statistics of Crime":—

"A 'Blue Book,' containing the Nineteenth Report of the Inspectors of Prisons in Great Britain is just published. The volume contains 247 pages of closely-printed numerals; and though it is not easy at first to extract the results from such an arithmetical mass, there are some points which are well deserving of serious consideration. The report gives a comparative analysis of the total number of prisoners tried at the assizes and sessions, the number of convictions and the summary convictions for every year from 1841 to 1853, both inclusive; and it is gratifying to find that, notwithstanding the increase of population, there has been a gradual, but sure, decrease in the number of crimes committed, and also a diminution in the magnitude of the offences. It will suffice, however, for our purpose to take the relative amount of crimes committed in the years 1852 and 1853, the last period to which the returns are made up.

"The numbers for trial, or tried at assizes or sessions in 1852, were 27,350; in 1853, 26,804; being a decrease of two per cent. The convictions, as compared with the committals, are identical in their proportions, being in the year 1852 77·3, and in 1853 77·0 per cent. The decrease in the number of summary convictions is still more cheering. The total number of summary convictions in 1852 was 76,547, and in 1853 only 71,850, showing a decrease of 4967 or 6·5 per cent. These convictions have in the returns been classified under different heads, which renders their application easier. First on the list stand offences against the game laws, and here it is most satisfactory to witness a great improvement. These game laws have been by many considered the curse of the country, and at one period did more to fill our gaols than anything else. Formerly, to shoot a partridge was in the eye of the law as bad as committing a burglary or a highway robbery. A young countryman who, on the impulse of the moment, shot a pheasant, very soon found himself a convicted felon with the worst possible characters for his companions; and he from that moment became a lost man. To have been an inmate of a felon's prison was like the brand upon Cain; and henceforth he became a wanderer on the face of the earth, his hand against every one, and every one's hand against him. Such were the effects of the game laws but a few years since; and, although the laws remain the same, yet the landed aristocracy find it is inexpedient to enforce them rigidly. The effect of this forbearance has been to reduce the number of commitments for offences against the game laws from 3525 in 1852 to 2671 in 1853—a decrease equivalent to 24·2 per cent.

"There has also been a marked diminution in the number of offences committed against the revenue laws, the numbers having fallen from 987 in 1852 to 768, or 22 2 per cent. This diminution in crimes against the revenue may probably be attributed to the decrease in the amount of smuggling, arising from the operation of free trade. There appears to be a diminution in illicit distillation. In offences against the bastardy laws there has likewise been a decrease in the year of 23·9 per cent; the number of convictions in 1852 having been 1097, and in 1853 only 835. Whether this arises from a higher tone of morality, or from the prosperous condition of the labouring population, which tends to swell the number of marriages, is not determined in the returns; but the latter conclusion would appear to be the correct one. The number of offences against the Vagrant Act has also decreased 22·8 per cent., the number of offences in the two years 1852-3 being respectively 20,314 and 15,685. The diminution in this class of offences is clearly attributable—first, to the abundance of employment, and, secondly, to the calling out of the militia, which absorbed a considerable portion of the idle and worthless population.

"Persons seem in the year 1853 to have been much more amicably disposed than in the previous year, as we find there was a diminution of 16 per cent. in the number of offences committed against the Malicious Trespass Act. Neither do the people appear to have been as pugnacious as usual, or perhaps the war drew off much of the hot blood of the country, as there is a diminution of 2·1 per cent. in the number of convictions for assault. There has also been a diminution of 3·5 per cent. in the number of persons summarily convicted as being reputed thieves.

Thus much on the fair side of the picture. On the other hand, the number of offences committed by military prisoners increased 395·2 per cent., the number of convictions having been only 42 in 1852, and 208 in 1853. This is, however, not at all extraordinary, owing to the militia having been embodied during the latter year, and considering the number of men raised, the actual amount of convictions against military prisoners must be regarded as small. In offences under the Larceny Act there has been a slight increase in the number of convictions to the extent of 1·1 per cent. But under the Police Act there has been an increase of 29·8 per cent., an unusually large amount, as regards which the report gives no information. In all other offences, not included under any of the above heads, the number of convictions in 1852 were 17,767, and in 1853 18,014 in this class. However, the convictions under the Militia Act included in this class, amounting to 244, being deducted, reduce the numbers to 17,780, exhibiting a very slight increase upon the preceding year. In offences committed by juvenile offenders it is gratifying to notice a diminution. The number of prisoners under the above age for trial, or tried at the assizes or sessions in 1852 were 2222, in 1853, 2105, being a decrease of 117, or 5·2 per cent. The summary convictions were in 1852 9599, and in 1853 9348, showing a decrease of 2·6 per cent.

Whilst, however, on the whole, it is gratifying to find that there has been such a marked diminution in the amount of crime, it is to be regretted that there is no evidence of education having made much progress, as it is shown that from 1850 to 1853 inclusive, giving the proportion which the uneducated criminals bear to the total numbers, that scarcely any improvement has been observed in this most important branch of the question. Thus, of the total number of criminals in 1850, there were 94·8 who possessed little or no instruction; in 1851, the numbers were 95·1; in 1852, 95·4; and in 1853, 95·3 per cent. There is, therefore, plenty of scope for the labours of all those who are interested in the improvement of the people. Such are the main conclusions arrived at in this report of the Prison Inspectors."

One of the wise men of old used to say that the sum of virtue was comprised in the two words, *bear* and *forbear*. We are all so liable to make mistakes in word and action, that whenever an error of speech or conduct can possibly be overlooked and forgotten, the sooner it is done the better. The forbearance in enforcing the Game Laws against offenders under this head, is an instance in point. The next means of lessening crime is that of education. The number of children that wander about the streets of every town in rags and ignorance, infamy and want, is too apparent not to have attracted the attention of the most indifferent observer. These little urchins ought to be provided for and educated in some manner, however defective. A little education and a little discipline would be better than none; a great point would be gained if they were taught how to keep themselves clean, and, above all, enabled to do so. They might be taught something; they would only be too ready to learn that something, whatever it might be; and who doubts but that they would be but too glad to keep their little hands from picking and stealing, if only some of the "dons" would assist them in doing so. As it is, they grow up in darkness and destitution: their only trade is that of providing for themselves by petty larceny, for which they are convicted before the bench, sentenced to hard labour, and sent to the House of Correction, where they associate with others like themselves, and from whence, at the end of their term of "durance vile," they come forth hardened in shame and confirmed in their evil propensities. The end of it is, that they have the ignoble satisfaction of furnishing statistics of crime to be duly laid before honourable members and the public in general, in the shape of a Blue Book of three or four hundred pages of questions and answers, to be eventually legislated upon *pro bono publico* in its proper order, time, and place. Much, if not all, of this cumbrous machinery might be effectually dispensed with by looking after the numberless outcasts of society, and protecting them, not so much for their sakes as for our own.

We are compelled this quarter to postpone our critical analysis of one of the most recent essays on insanity in relation to crime.* We subjoin the following *resumé* of the conclusions to which the writer has arrived:—

"*That the insane man is responsible for his insanity when the latter has been produced or occasioned by circumstances over which he either has or had control.*

"*The insane man is not responsible for his insanity when the latter has been occasioned by circumstances over which he has or had no power of control.*"

"1. *The insane man is responsible for actions which present no discoverable relation to the definitely morbid condition of his mind.* In other words, the insane man is responsible for those actions which cannot be traced to his insanity.

"2. *The insane man is responsible for his criminal actions when there is no evidence of insanity beyond that of the act performed.* It may be questioned in many cases whether the individual is insane at all. But it is assumed that the plea is raised, and that the case is considered one of so-called 'insane impulse.'

* "Criminal Lunatics: Are they responsible? Being an Examination of the Plea of Insanity, in a Letter to the Right Hon. the Lord Chancellor." By J. Russell Reynolds, M.D. London: 1856.

"3. *The insane man is not responsible for those actions which are the direct result or expression of his insanity.* The presence of a distinct delusion or erroneous belief (!) which the individual cannot correct, but upon the faith of which he acts, and it may be criminally, is sufficient to establish his irresponsibility.

"4. *The insane man is not responsible for criminal actions when without any distinct or persistent delusions the whole tenor of his mind is deranged.*"

The next point raised is this: "Is the insane man, when responsible, as much so, or equally responsible with the sane?" The following are the propositions:—

"1. *The insane are as responsible as the sane for actions committed through insanity which they have voluntarily brought upon themselves.*

"2. *Under all other conditions of insanity there is some diminution of responsibility*, inasmuch as the benefit of doubt should be extended to those who have this claim upon the mercy of society. But granting that responsibility is diminished, no definite lines can be drawn for fixing its amount, except from a consideration of the requirements of particular cases."

We extremely regret that Dr. Reynolds, in flapping his newly-fledged psychological wings, should have authoritatively sanctioned doctrines so repugnant to reason, so opposed to sound mental pathology, and so contrary to the principles of justice and humanity. It is indeed a sad but significant sign of the times to find an educated physician living in the present enlightened epoch gravely enunciating, in a pamphlet addressed to the highest judge in the land, that an insane person should be considered legally responsible for offences committed against the state, if the insanity "*has been occasioned by circumstances over which he either has or had control;*" and that "*the insane are as responsible as the sane for actions committed through insanity, which they have voluntarily brought upon themselves.*" "They" (continues Dr. Reynolds, speaking of lunatics legally responsible for their conduct in consequence of the insanity being self induced) "have possessed and employed the faculty of choice; they have recognised possible advantages and possible evils; they have preferred self-gratification to every other good, and if by a course of this description *they have become criminal*, they are justly responsible for the crime, and as responsible *as if they were sane* at the time of its commission."

"A Daniel!—a second Daniel!—come to judgment."

What *are* the judges to think of crude, unphilosophical, and inhumane opinions like these, if they can properly be received as an embodiment of the sentiments of the British psychological physician? God forbid that such doctrines should ever fasten themselves upon the public, professional, and judicial mind.

Is there a type of mental derangement that may not, agreeably to Dr. Reynolds's signification of the term, have "been occasioned by circumstances over which he (the lunatic) either has, or had, control?" A man is exposed to a heavy domestic calamity, the result, perhaps, of reckless speculations on the Stock Exchange. In a few hours he is reduced from affluent to indigent circumstances. Prostrated by the affliction, his bodily health becomes seriously impaired, the mind succumbs, and insanity, in its most serious form, develops itself. This man may have been guilty of great improvidence and indiscretion, and have shown a culpable want of judgment, common sense, and ordinary forethought. He has ruined himself by his own voluntary acts of folly, and the sad consequences to his body and mind have clearly been "occasioned by circumstances over which he has had control." Would Dr. Reynolds—kind and humane physician we believe him to be—witness without emotion this man dangling from the end of a rope, in the convulsive agonies of a painful death for the amusement of some 20,000 blackguards, if he should happen, in a paroxysm of delirium, connected with his "voluntarily induced insanity," to murder his wife or child? The principal cause of insanity that prevails to so great an extent among the pauper portion of our population is, beyond all doubt, *intemperance*. Dr. Reynolds could easily satisfy his mind upon this point, if he will take the trouble of visiting any one of our County Lunatic Asylums. The insanity of many of the poor unhappy beings confined in these institutions is clearly "occasioned by circumstances over which they had control"—they have *voluntarily* drank of that which

"Takes the reason prisoner,"

and, therefore, agreeably to Dr. Reynolds's psychological test, "they are as responsible *as the sane* for their actions," and should be hanged, or otherwise severely punished, if they in a state of profound dementia, idiocy, or maniacal excitement, committed the crime of murder. When alluding to the subject of self-created insanity, in our comments on the case of Mrs. Brough, we remarked:—

"The same cause (intemperance) is in operation but to a limited extent in the middle and upper classes of society; but insanity may often be traced to a criminal indulgence in depraved habits and vicious thoughts, to reckless and unprincipled conduct; to long indulged self-will; to a censurable neglect of the cultivation of habits of *self-control*; to an utter disregard of all mental discipline and training.

"If we are justified in considering every person accountable and amenable to punishment, whose insanity can be clearly traced *to self-created causes*, where are we to draw the line? The man who, as the result of a series of debaucheries, *voluntarily* drinks himself into a state of furious delirium, is, as long as that delirium continues, *non compos mentis*, and he ought not to be considered accountable for any act committed during his paroxysm of *self-induced* and temporary insanity. We may regret that there should not exist, for cases like these, a secondary form of punishment, which, if judiciously awarded, might prevent much of the deplorable misery we are daily compelled to witness in social life; but until our criminal code has undergone material alterations, it is not for us to draw refined distinctions, and say that one class of insane persons should not escape the legal penalties to which they have by their conduct exposed themselves, simply because their mental disease can be traced to intemperance, unbridled passion, or unchecked vicious impulses and thoughts; and a different class of the insane, whose sad condition has originated from causes quite out of their own control, should entirely escape punishment and censure."*

If we were to carry into practical operation Dr. Reynolds's theory, our courts of law would necessarily become arenas for the discussion of subtle points of psychology, practical medicine, and therapeutics; for in cases in which the plea of insanity is urged as an excuse for crime, the question for the consideration of the jury would not be, is the prisoner sane or insane, responsible or irresponsible, but was the alleged insanity occasioned by circumstances which he could or should have controlled; could he have warded off his attack by avoiding all undue excitement, anxiety of mind, sudden mental emotion, or by having paid stricter attention to dietetic rules, to the state of his stomach, digestive organs, and liver? It would be the duty of the jury to consider, whether the unhappy culprit's insanity had not resulted from his having voluntarily neglected to comply with certain organic conditions of mental health. If this could be established to the satisfaction of the court, Dr. Reynolds's test of responsibility in connexion with crime and insanity would be demonstrated, and Calcraft would have an opportunity of exhibiting his skill by hanging the poor imbecile, or idiot,

> "His gallant bark of reason wreck'd,

> A poor quench'd ray of intellect,

> With slobber'd chin, and rayless eye,

> A mind of mere inanity."

Let Dr. Reynolds ponder well upon this subject—let him seriously reconsider this important matter, and he will, we feel assured, be the first to eschew doctrines alike abhorrent to the feelings of a Christian, repulsive to men of science, and subversive to the first principles of sound judicial psychology.

* "Psychological Journal" for Oct., 1854.

